# Optimization of operating conditions for the catalytic alcoholysis of waste PET for the synthesis of BHET by sunflower seed husk matrix materials†

**DOI:** 10.1039/d4ra07206e

**Published:** 2025-01-02

**Authors:** Linlin Zhao, Guoliang Shen, Ruiyang Wen, Tiejun Xu, Sijin Jiang, Xiaocui Wang, Haichen Wang

**Affiliations:** a School of Petrochemical Engineering, Shenyang University of Technology Liaoyang 111003 China 13869119262@163.com petrochem@126.com Wenruiyang6@163.com xtjltlt@126.com 2093894641@qq.com 2668991705@qq.com 2572175546@qq.com

## Abstract

A sunflower seed shell matrix catalyst (SMS-750) was prepared from sunflower seed shell waste by pretreatment and pyrolytic carbonization. A series of characterization analyses showed that the prepared catalyst was rich in Ca, Mg, K, and other mineral elements and mainly existed in the form of metal oxides. SMS-750 was used to catalyze the glycolysis of waste PET, and the main factors affecting the BHET yield were screened out by a one-way experimental design, and then the BHET yield was used as the response value, and the response surface method was used to design and analyze the effects of the respective variables and their interactions on the degradation of PET according to the principle of Box–Behnken central combinatorial design. The optimum reaction conditions were optimized using the predicted quadratic regression model with a reaction temperature of 185 °C, a reaction time of 4.9 h, a catalyst dosage of 0.89%, and an ethylene glycol dosage of 14.6 ml, and the BHET yield of 79.57% was obtained under these conditions. The degradation of waste PET by SMS-750 prepared in this paper provides a useful reference for the resourceful use of waste and sustainable development.

## Abstract 2

A biomass-based catalyst (SMS-750) was prepared from waste sunflower seed husk to catalyze the alcoholysis of waste PET to obtain a high value-added product (BHET).

## Graphical abstract

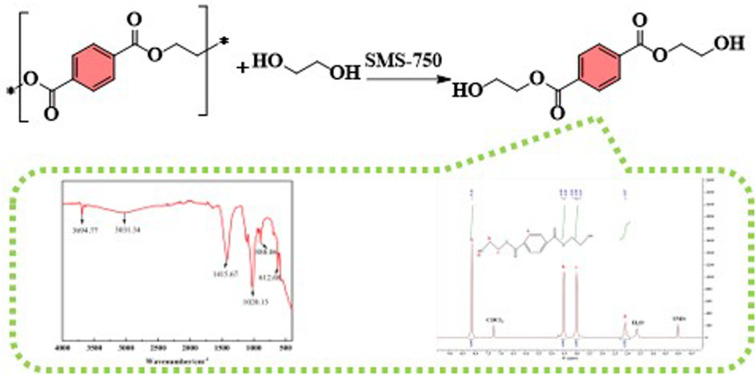

## 1. Introduction

Polyethylene terephthalate (PET), as an important thermoplastic polyester material, comes from fossil resources.^1,2^ PET has excellent properties such as good tensile strength, chemical resistance, transparency, processability, and thermal stability, and is therefore widely used in food packaging, electrical and electronic appliances, machinery and equipment, automotive parts, films and sheets, and so on.^3^ In recent years, with the continuous progress of PET production technology and the continuous growth of market demand, PET production capacity has shown a steady upward trend. According to statistics, the global bottle-grade PET production capacity has grown to 34.87 million tonnes from 27 million tonnes in 2014 to 2022, and it is expected that by 2050, 12 billion tonnes of plastic will be thrown into landfills and nature. However, due to the excellent chemical stability of PET, it is difficult to degrade in the natural environment, and a large amount of waste PET is decomposed into microplastics in the soil and flows into lakes and oceans, which poses a serious hazard to people's living environment and health.^4,5^ In addition, virgin PET is a petroleum-based material, but the PET recycling rate is not high, resulting in a waste of fossil resources.^6,7^ To save fossil resources and protect the environment, there is an urgent need to develop a PET recycling process.^8–10^ Today, the treatment methods of waste PET are mainly divided into physical recycling methods and chemical recycling methods.^11–13^ Physical recycling is simple and inexpensive, but it cannot completely remove the impurities in PET, thus affecting the quality of recycled products. Chemical recycling converts waste PET into monomers or oligomers and other chemical substances through chemical reactions, and then re-polymerizes or uses them, which can realize the complete recycling and reuse of waste PET, and has a high resource utilization rate and environmental friendliness.^14–16^ Among them, alcoholysis is considered to be an ideal method for PET degradation due to its mild reaction conditions, low solvent volatilization, and high product purity. Alcoholysis using ethylene glycol as a solvent is called glycolysis.^17,18^ The main product of glycolysis is ethylene terephthalate (BHET), which can be re-synthesized into PET by polymerization.^19^ The reaction rate of glycolysis without a catalyst is very low. Metal salts,^20^ metal oxides, low eutectic solvents (DES)^8,21–24^ and ionic liquids^25–28^ have been developed to catalyze the degradation of PET. Although these catalysts have good catalytic effects, they also have the disadvantages of harsh reaction conditions, low monomer yield, and difficult catalyst separation.

Currently, biomass catalysts have been developed as a green alternative to traditional metal catalysts. Isti Yunita *et al.* extracted calcium oxide (CaO) from ostrich eggshells, and seafood shell biomass as raw material to catalyze post-consumer PET bottles and examined their catalytic activity. The results of the study showed that ostrich eggshell by-products of CaO have the advantages of low cost, environmentally friendly, and high product yields.^29^ To the best of our knowledge, there is no report on the production of metal oxides from sunflower seed shells as catalysts for PET glycolysis. In this paper, SMS-750 was prepared from sunflower seed husk and applied to PET glycolysis. In order to better understand the catalytic parameters, the main factors such as alcoholysis temperature, glycol dosage, catalyst dosage and alcoholysis time were firstly screened by one-way experiments, and then the best reaction conditions were determined by optimization using response surface experiments. In addition, the alcoholysis products and catalysts were characterized and analyzed. The development of catalytic degradation of waste PET by biomass catalysts will help promote the green transformation of the plastics industry. By reducing the environmental pollution and waste of resources caused by waste plastics, the sustainable development of the plastics industry and the goal of “double carbon” can be achieved.

## 2. Materials and methods

### 2.1 Materials

Waste sunflower seed husk, agricultural waste material; waste polyester, 0.3 × 0.3 cm, mineral water bottle; ethylene glycol, anhydrous ethanol, analytically pure, Tianjin Beichen Founder's Reagent; double-distilled water, 0.5% NaOH solution, homemade in the laboratory.

### 2.2 Equipment and instruments

Fourier Infrared Spectrometer (FT-IR), TENSOR II, Bruker Technologies, Germany; Nuclear Magnetic Resonance Hydrogen Spectroscopy (^1^H-NMR), AVANCE, Bruker Technologies, Germany; Thermogravimetric Analyser (TG), HCT-1, Hengjiu Scientific Instrumentation Factory, Beijing, China; Scanning Electron Microscope (SEM), Apreo 2C, Thermo Fisher Scientific; X-Ray Energy Spectroscopy (EDS), XFlash 6130, Oxford Instruments, UK.

### 2.3 Preparation of SMS-750 catalysts

**Fig. 1:**
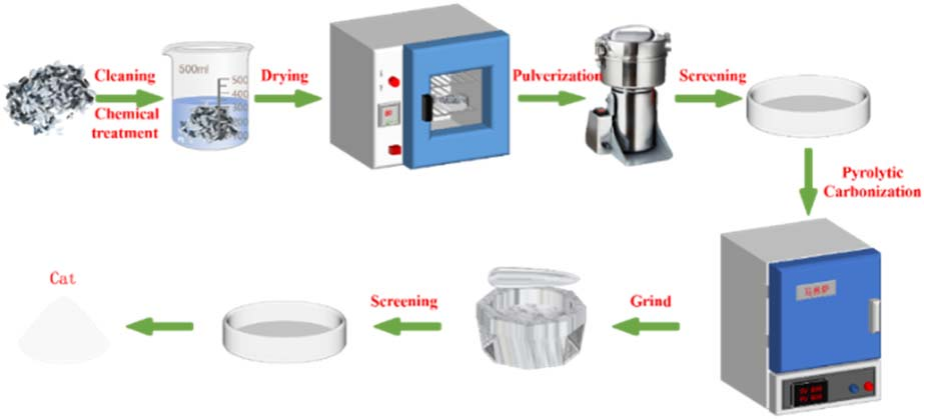
Schematic diagram of sunflower seed shell matrix catalyst preparation.

Firstly, the edible sunflower seeds were repeatedly washed with double-distilled water to remove surface impurities. In order to further remove the residual lipid components in the sunflower seed hulls, the hulls were soaked in 0.5% NaOH solution for 2 h, washed, and then dried at 80 °C for 24 h. Subsequently, the dried sunflower seed hulls were crushed in a pulverizer into homogeneous particles and screened with a standard 100-mesh sieve. The sieved sunflower seed husk powder was then placed in a crucible and then roasted in a muffle furnace preheated to the specified temperature for 2 h. After the temperature was reduced to room temperature, the calcined solid powder was finely ground in a mortar and pestle to obtain a more homogeneous particle size distribution. Finally, in order to ensure the consistency and suitability of the resulting catalyst size, the ground samples were sieved using a 200-mesh standard sieve, *i.e.*, the solid powder obtained was SMS-750 (Fig. 1).

### 2.4 SMS-750 catalyzed glycolysis of waste PET

**Fig. 2:**
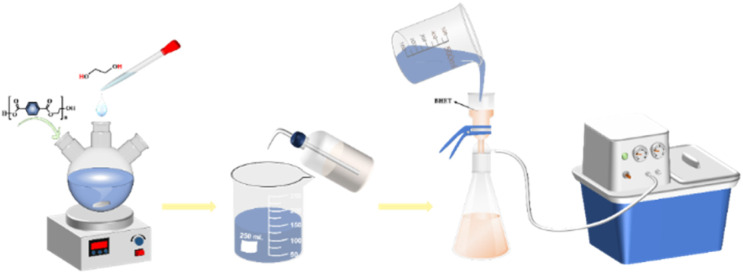
Schematic diagram of SMS-750 alcoholysis of waste PET.

Weigh 3.0 g of waste PET bottle flakes in a three-necked flask, add the prepared sunflower seed husk matrix catalyst and ethylene glycol solution according to a certain ratio, stirring, heating, heating to a set temperature, and then react for a certain length of time, you can get the impurity-containing BHET. When the reaction was finished, the reaction solution was transferred to a beaker and distilled water was added to 60 ml while it was still hot, and the temperature in the beaker was 90 °C when the first filtration was carried out using a filtering flask with the aim of filtering out the undepolymerized PET bottle flakes. The product from the first filtration was then filtered for the second time by adding distilled water to 90 ml and controlling the temperature at 65 °C, to separate the trimers. Further to remove the dimer, the product after the second filtration was added with distilled water to 120 ml and the temperature was controlled at 45 °C and then filtrated to obtain the BHET solution. The filtrate was cooled at 5 °C for 12 h. White needle-like crystals appeared in the beaker. Finally, after two filtrations, the white needle-like crystals were placed in a vacuum desiccator at 80 °C for 12 h to obtain the product BHET (Fig. 2).

Where the BHET yield is calculated in eqn (1) as follows:1
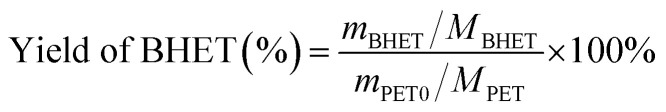
where *m*_BHET_ is the weight of BHET crystals collected in grams, *M*_BHET_ is the molecular weight of BHET (254 g mol^−1^), *m*_PET0_ is the initial weight of PET in grams and *M*_PET_ is the molecular weight of the PET repeat unit (192 g mol^−1^).

### 2.5 Testing and characterisation

XRD was used to analyze the crystal structure of the catalysts under the following conditions: voltage 40 kV, current 40 MA, scanning speed 10° min^−1^, test range 5°–90°, step size 0.02°; SEM was used to characterize the surface morphology of the catalysts under the following conditions: a small amount of the sample was adhered to conductive adhesive and sprayed with gold, with a pressurized voltage of 30 kV and a resolution of 1.4 nm, with a maximum multiplicity of about 100 000; the functional groups of the catalysts and degradation products were analyzed by FTIR. The functional groups of the catalyst and the degradation products were analyzed by FTIR test, under the following conditions: the samples were mixed and ground with KBr, then dried and pressed into tablets, with a scanning range of 400–4000 cm^−1^; the proportion of different types of hydrogen atoms in the degradation products was quantitatively determined with the help of ^1^H-NMR test by analyzing the peak area, and the following conditions were adopted: a sample of about 5 mg was taken and completely dissolved in CDCl_3_. The catalyst material was characterized for thermal stability using the TG test, where the sample was heated from 20 °C to 800 °C at a rate of 10 °C min^−1^ in the N_2_ atmosphere.

### 2.6 Response surface optimisation experiments

Based on a one-way parallel test, the response surface test was designed by Design Expert 13.0 software using the alcoholysis temperature, alcoholysis time, EG dosage, and SMS-750 dosage as independent variables, and the degradation product BHET as the response value, the test was carried out under the conditions of the corresponding independent variables, and the response values were obtained and substituted into the software for the response surface analysis, and the results were discussed.

## 3. Results and discussion

### 3.1 Characterisation of catalysts and degradation products

#### 3.1.1 Thermogravimetric analysis of sunflower hulls

**Fig. 3:**
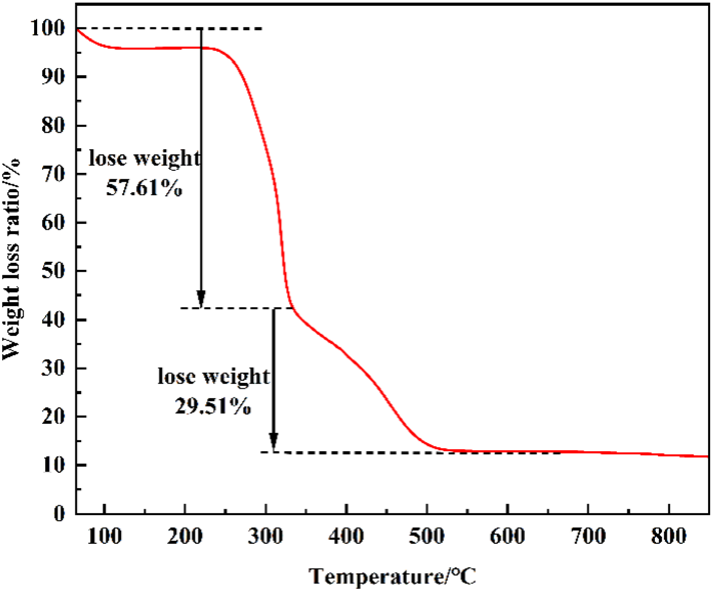
Thermogravimetric curve of sunflower seed husk.

In order to determine the variation of the composition of sunflower seed husk with different roasting temperatures, a certain amount of sunflower seed husk powder was taken for thermogravimetric analysis and the results are shown in Fig. 3. It can be seen that before 330 °C, the weight loss is about 57.61%, which is mainly caused by the loss of water and the mass of organic components in sunflower seed husk; the mass loss in the range of 330–600 °C is mainly attributed to the further pyrolysis of the remaining organic components in the residual charcoal, with a weight loss of 29.51%; the temperature continues to increase, and the weight loss is almost zero, which indicates that sunflower seed husk is roasted at greater than 600 °C after the weight loss was almost zero, indicating that the sunflower hulls had been constant after roasting at >600 °C. Based on the analysis of the effect of different calcination temperatures on the degradation yield, 750 °C was selected as the best calcination temperature for the catalyst, and the catalysts prepared at 750 °C were named SMS-750, based on which further research was conducted and discussed.

#### 3.1.2 SEM-EDS analysis of catalysts

**Fig. 4:**
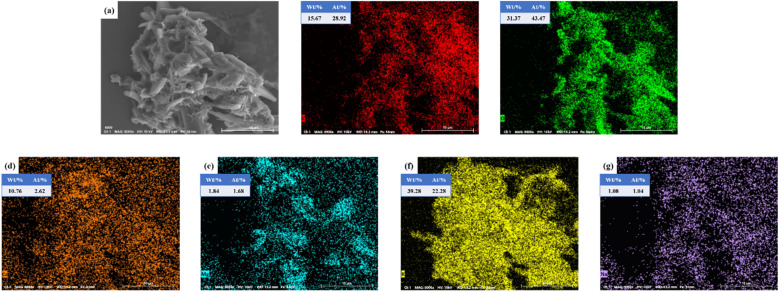
SEM photographs and EDS analysis of SMS-750.

The appearance morphology and elemental composition of SMS-750 were analyzed, and it can be seen from Fig. 4(a) that SMS-750 is mostly in the random stacking of irregular bricks with smooth surfaces and a small amount of microporous structure. According to the EDS of Fig. 4(b–g), the mass fractions of the elements K, O, C, Ca, Mg, and Na in SMS-750 were 39.28%, 31.37%, 15.67%, 10.76%, 1.84%, and 1.08%, and the elements were uniformly distributed on the catalyst surface.

#### 3.1.3 XRD analysis of catalysts

**Fig. 5:**
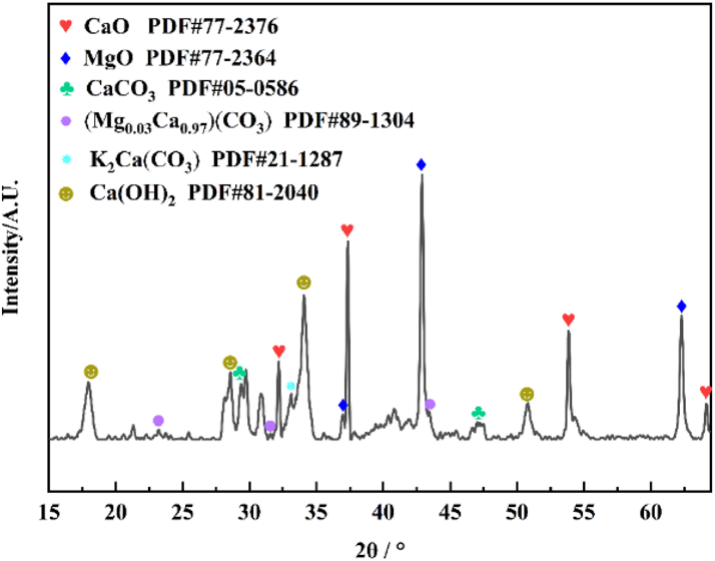
XRD diagram of SMS-750.

The associated XRD diffractogram in Fig. 5 depicts the crystalline structure of SMS-750. The presence of a crystalline phase is indicated by the presence of multiple peaks in the diffraction peaks centered at 2*θ*. The sharp peaks evident in the spectra indicate that the sample is well crystalline. The results show that the major compounds in SMS-750 are CaO, MgO, CaCO_3_, (Mg_0.03_Ca_0.97_)(CO_3_), K_2_Ca(CO_3_) and Ca(OH)_2_. It can be seen that SMS-750 is highly loaded with oxides and carbonates of K, Ca, and Mg, which have very strong basic sites, which is highly consistent with the results of EDS analysis.

#### 3.1.4 FT-IR analysis of catalysts

**Fig. 6:**
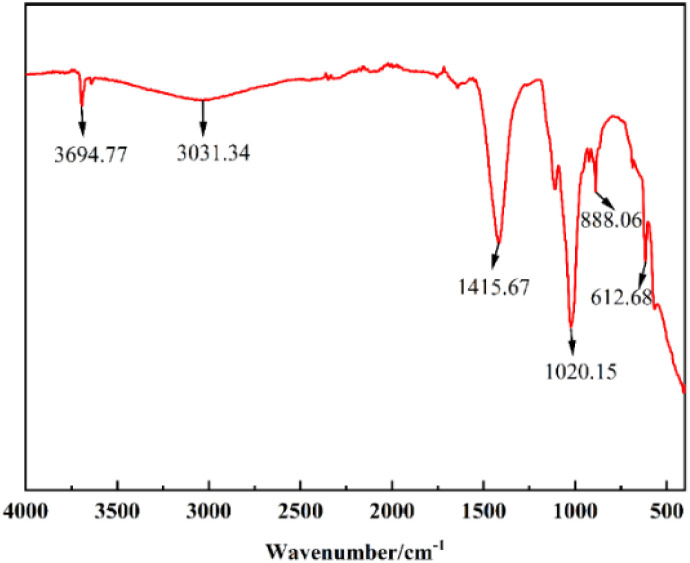
FT-IR diagram of SMS-750.

The infrared spectra are shown in Fig. 6. 3031.34 cm^−1^ the broad absorption peaks are due to –OH stretching vibration due to water absorption on the catalyst surface.^30^ The sharp bands at 3694.77 cm^−1^ are associated with the formation of basic groups attached to Ca atoms.^31^ The characteristic peaks at 1415.67 cm^−1^, 1023.15 cm^−1^, and 888.06 cm^−1^ correspond to the C=O stretching vibration, C–O stretching vibration and C–O bending vibration of CO_3_^2−^ respectively, which may be due to the absorption of CO_2_ from the air onto the surface of the metal oxides to form metal carbonates, thus suggesting the presence of Ca, Mg and K oxides in the catalyst.^32–36^ The absorption peaks of K–O and Ca–O stretching vibration are at 612.68 cm^−1^.^37,38^ The FT-IR results are in agreement with EDS and XRD data.

#### 3.1.5 FT-IR analysis of degradation products

**Fig. 7:**
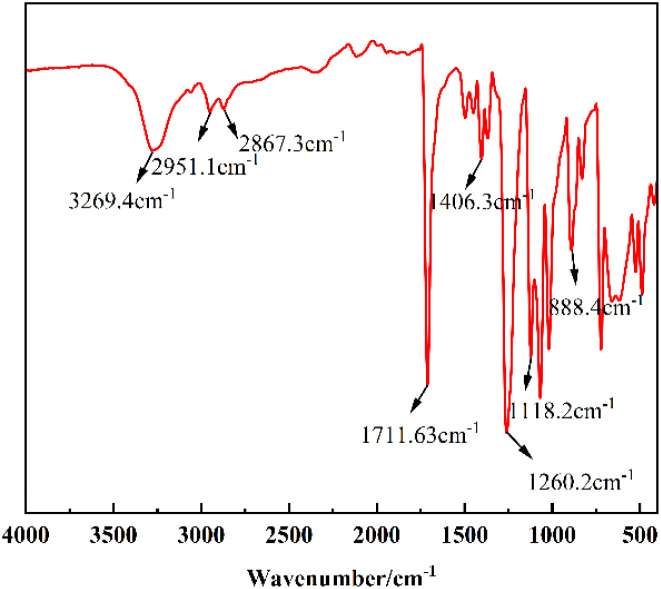
Infrared spectrogram of BHET.

The IR spectral characterization of the degradation products is shown in Fig. 7. It can be seen that the characteristic absorption peak at 3269.4 cm^−1^ corresponds to the –OH stretching vibration in the hydroxyethyl group, the infrared absorption peaks at 2951.1 cm^−1^ and 2867.3 cm^−1^ are the symmetric stretching vibration peaks of –CH_2_, the strong absorption peaks at 1711.63 cm^−1^ are related to the stretching vibration of C=O, the characteristic absorption peaks near 1406.3 cm^−1^ are the vibrational absorption peaks of the benzene ring backbone. The absorption peaks at 1260.2 cm^−1^ and 1118.2 cm^−1^ are the absorption peaks of the stretching vibration of C–O, and the in-plane bending vibration of the benzene ring is 888.4 cm^−1^, which in summary indicates that the IR spectra of the product are in agreement with those of BHET.

#### 3.1.6 ^1^H-NMR analysis of degradation products

**Fig. 8:**
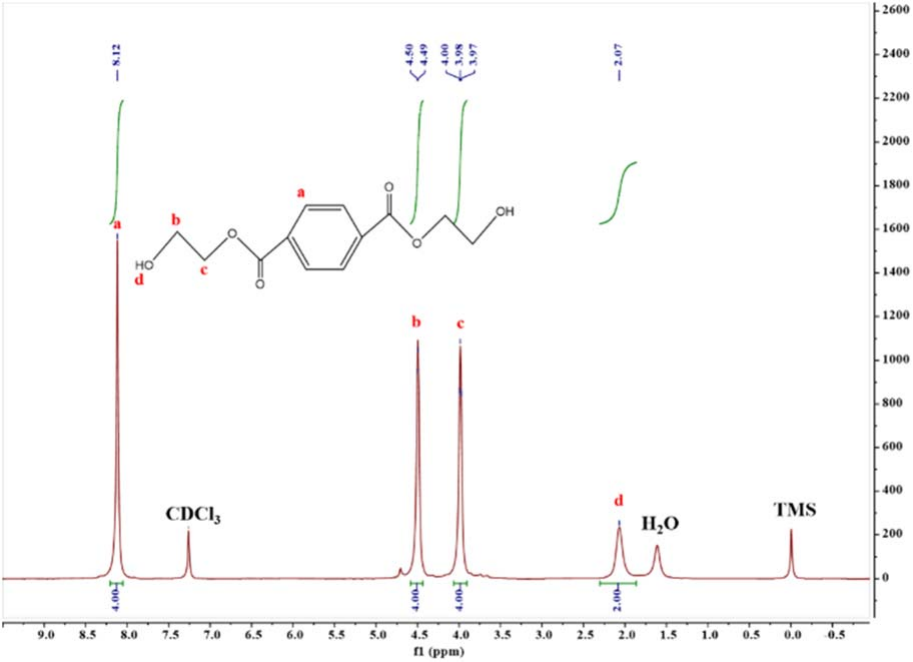
^1^H-NMR plot of BHET.

In order to further determine the structure of the degradation product, the alcoholysis product obtained by SMS-750 catalysis was characterized by ^1^H-NMR, and the results are shown in Fig. 8. The ^1^H NMR (400 MHz, CDCl_3_) *δ* 8.12 (s, 4H), 4.50 (d, *J* = 5.8 Hz, 4H), 4.06–3.91 (m, 4H), 2.07 (s, 2H), the ratio of the number of hydrogen atoms was calculated from the peak area, and the ratio of hydrogen atoms of the PET degradation product and the monomer BHET molecule matched, and combined with the analysis of the FT-IR spectra, it can be confirmed that the PET degradation product is BHET.

### 3.2 One-factor experiments on PET glycol degradation

**Fig. 9:**
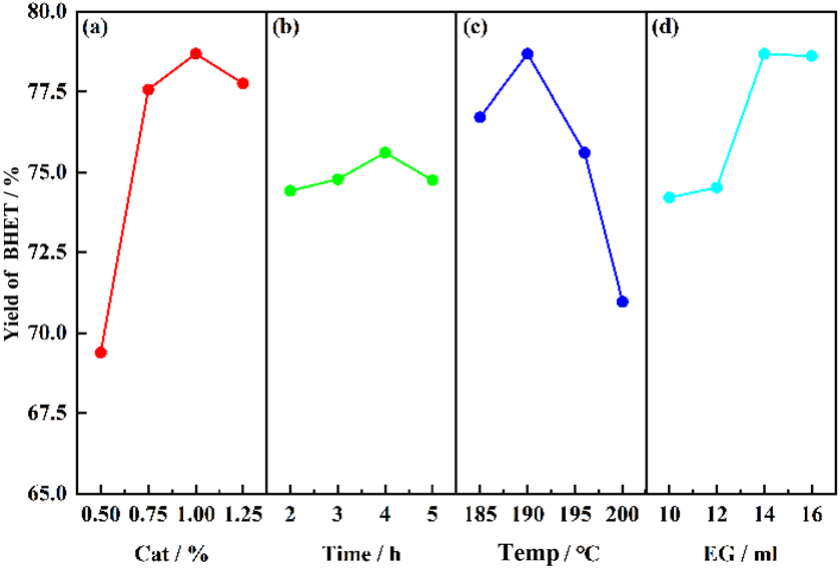
Effect of different factors on the yield of BHET.

The main influencing factors in the SMS-750 catalyzed PET alcoholysis process are catalyst dosage, alcoholysis time, alcoholysis temperature, and solvent dosage. The four factors were selected as the main influencing parameters for the one-way experiment, as shown in Fig. 9. It can be seen that when the catalyst dosage is 1%, the alcoholysis time is 4 h, the alcoholysis temperature is 190 °C and the solvent dosage is 14 ml, the BHET yield relatively reaches a higher value, which provides a coded level of central value for the establishment of the response surface below.

### 3.3 Response surface methodology experimental design and determination of optimization conditions

#### 3.3.1 Box–Benhnken experimental design

According to the Box–Behnken central combination design principle, a 4-factor, 3-level response surface analysis experiment was designed using the alcoholysis temperature (*A*), alcoholysis time (*B*), EG dosage (*C*), and SMS-750 dosage (*D*) as the independent variables, and the degradation product, BHET yield (*Y*), as the response value, and the levels and codes of the experimental factors are shown in Table 1. A total of 29 sets of trials were run for the response surface design, details of which are given in the ESI, Table S1.†

**Table 1:** Response surface test factor and level design

Independent variable	Unit	Coded levels		
		−1	0	1
*A*: alcoholysis temperature	°C	185	190	195
*B*: alcoholysis time	h	3	4	5
*C*: EG dosage	ml	12	14	16
*D*: SMS-750 dosage	%	0.75	1.00	1.25

#### 3.3.2 Analysis of variance and regression modeling

Multiple regression was fitted to the experimental data using Design-Expert software to obtain the quadratic fitted regression eqn (2).2
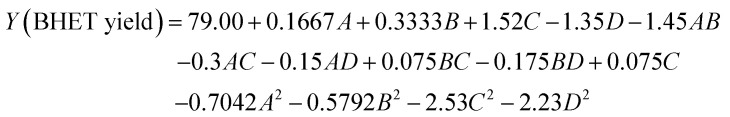


The simulated regression equations were analyzed by ANOVA and tested for significance and the results are shown in Table 2.

**Table 2:** Results of *Y* regression analysis^a^

Source	Sum of squares	Degrees of freedom	Mean squares	*F*-value	*P*-value
Model	122.50	14	8.75	3.77	0.0092
*A*	0.3333	1	0.3333	0.1435	0.7105
*B*	1.33	1	1.33	0.5740	0.4612
*C*	27.60	1	27.60	11.88	0.0039
*D*	21.87	1	21.87	9.42	0.0083
*AB*	8.41	1	8.41	3.62	0.0778
*AC*	0.3600	1	0.3600	0.1550	0.6997
*AD*	0.0900	1	0.0900	0.0387	0.8468
*BC*	0.0225	1	0.0225	0.0097	0.9230
*BD*	0.1225	1	0.1225	0.0527	0.8217
*CD*	0.0225	1	0.0225	0.0097	0.9230
*A* ^2^	3.22	1	3.22	1.38	0.2589
*B* ^2^	2.18	1	2.18	0.9367	0.3495
*C* ^2^	41.49	1	41.49	17.86	0.0008
*D* ^2^	32.23	1	32.23	13.88	0.0023
Residual	32.52	14	2.32		
Lack of fit	30.24	10	3.02	5.30	0.0610
Pure error	2.28	4	0.5700		
Cor total	155.02	28			
*R* ^2^	0.7902		C.V.%	1.99	
Adjusted *R*^2^	0.5805		Adeq. precision	7.1922	

a 0.01 < *P* < 0.05, significant difference; *P* < 0.01, highly significant difference.

As shown in Table 2, the model *P* = 0.0092 < 0.01, indicating that the model has good overall significance, high model confidence, and accurate simulation. According to the magnitude of the *F*-value, we can judge the significance of the four factors on the model in the following order: EG dosage > SMS-750 dosage > alcoholysis time > alcoholysis temperature. The misfit term of the model *P* = 0.0610 > 0.05 indicates that the misfit of the response values is not significant and the model can reflect the relationship between the independent variables and the response values better. The correlation coefficient of the model *R*^2^ = 0.7902, the adjusted coefficient of determination *R*^2^_Adj._ = 0.5805, and the coefficient of variation C.V. = 1.99% < 10% indicate that it has a sufficiently strong signal and the model is ideal. In conclusion, the simulation is accurate and reliable for optimizing and analyzing the predicted test conditions for SMS-750 catalyzed glycolysis PET.

#### 3.3.3 Analysis of interactions

In order to visualize the effect of the interaction between four factors, namely, alcoholysis temperature (*A*), alcoholysis time (*B*), EG dosage (*C*), and SMS-750 dosage (*D*), on the BHET yield (*Y*), three-dimensional response surface and plane contour plots of the relationship between the factors and the response values were plotted using Design Expert. The slope of the surface of the three-dimensional plot can reflect the influence of the factors on the response value, and the steeper the slope, the greater the influence of the factor on the response value; the strength of the interaction between the factors is reflected by the shape of the contour lines.

**Fig. 10:**
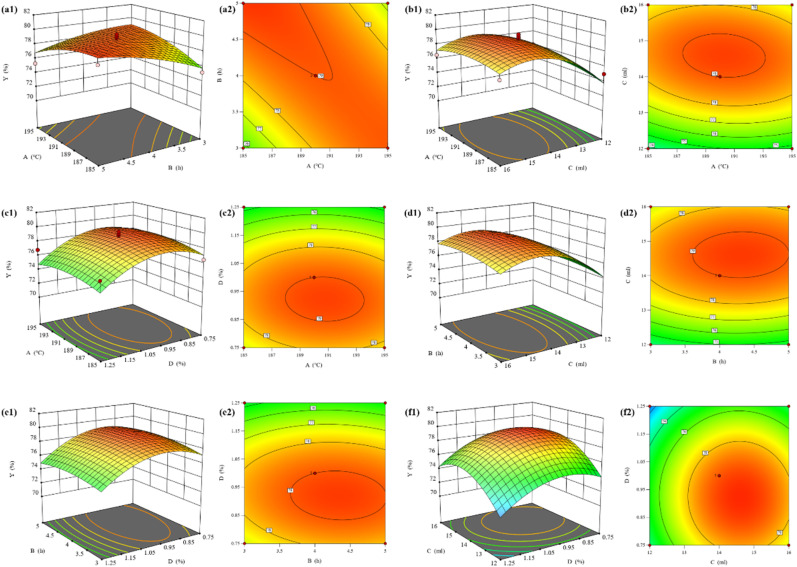
Stereo response surface and contour plots of response surface optimized BHET yields.

Combined with Fig. 10(a1) and 10(a2), it can be seen that the alcoholysis temperature and alcoholysis time have a more significant effect on the model and there is good interaction between them; combined with Fig. 10(b1) and 10(b2), it can be seen that the EG dosage has a greater effect on the BHET yield, which is shown to increase and then decrease, which is in line with the results of the one-way test; combined with Fig. 10(c1) and 10(c2) can be seen that the effect of SMS-750 dosage on BHET yield is larger compared to the alcoholysis temperature, and the slope is steeper; combined with Fig. 10(d1) and 10(d2), it can be seen that the effect of EG dosage on BHET yield is larger compared to the alcoholysis time, and the contour lines are elliptical, which indicates that the interaction between the alcoholysis time and the EG dosage is significant; combined with Fig. 10(e1) and 10(e2), it can be seen that the effect of SMS-750 dosage on the BHET yield was larger, which showed that it increased first and then decreased, which was consistent with the results of the one-way test; from Fig. 10(f2), it can be seen that the contour lines of the EG dosage and SMS-750 dosage were approximately circular, which indicated that the interaction between the two was not significant.

After the response surface analysis and the prediction of the regression model, the optimal reaction conditions for the alcoholysis of waste PET with SMS-750 were: alcoholysis time of 4.9 h, alcoholysis temperature of 185 °C, SMS-750 dosage of 0.89%, and EG dosage of 14.6 ml, and the simulated prediction of the BHET yield under this condition was 79.82%. Validation experiments were carried out for the above optimal conditions, and the final BHET yield obtained was 79.57%, which was similar to the predicted value, indicating that the use of this model is reliable.

### 3.4 Effect of repeated use of SMS-750

**Fig. 11:**
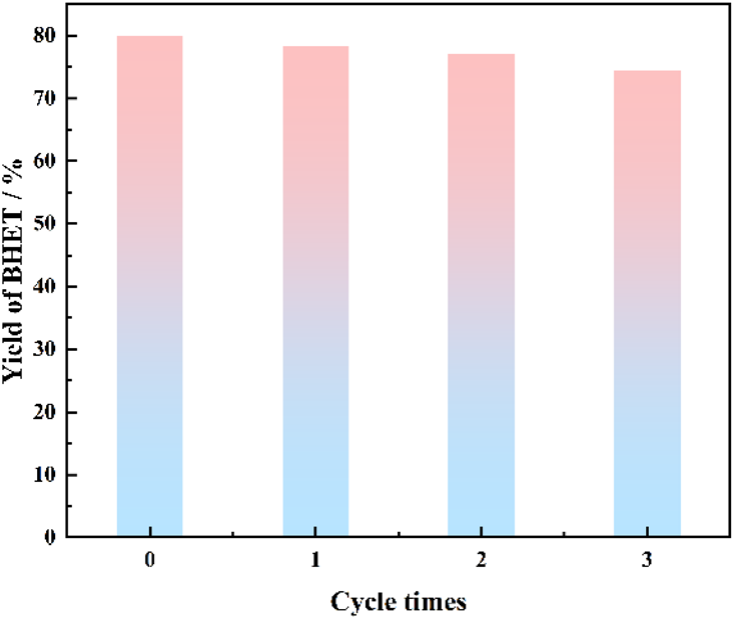
Effect of catalyst recycling.

The reaction was carried out under the conditions of alcoholysis temperature of 185 °C, alcoholysis time of 4.9 h, catalyst dosage of 0.89%, and ethylene glycol dosage of 14.6 ml. After the reaction, the catalyst was separated, washed, and dried while it was still hot, and reused several times to study the service life of the catalyst, and the results are shown in Fig. 11.

From Fig. 11, it can be seen that there was a slight decrease in the yield of BHET, which was mainly due to the small diameter of the prepared particles of SMS-750, and there was a slight loss of some catalyst dissolved in the reaction solution. The yield of BHET was still above 70% after three reuses. Therefore, the biomass catalysts prepared in this study have high catalytic activity and stability and can be reused many times.

### 3.5 Kinetic analysis of PET glycol depolymerization catalyzed by SMS-750

**Fig. 12:**
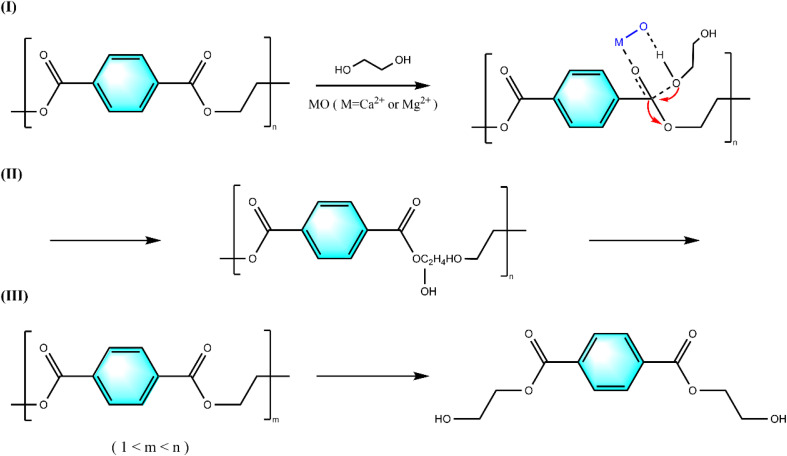
Reaction mechanism of PET alcoholysis catalyzed by SMS-750.

Based on the experimental results, Fig. 12 proposes a possible reaction mechanism for the catalytic alcoholysis of PET by calcium-magnesium oxide: (I) calcium-magnesium oxide is a solid base, whose active base position activates the hydrogen on the hydroxyl group of the ethylene glycol, which makes the oxygen on the hydroxyl group negatively charged and thus nucleophilic, and it is more likely to attack the electropositive carbonyl carbon atoms of the PET and undergo a nucleophilic addition reaction, which is more likely to attack the electropositive carbonyl carbon atoms of PET and undergo a nucleophilic addition reaction, and then the catalyst forms a six-membered ring transition state structure with the carbonyl group in the PET chain segments and the hydroxyl group in ethylene glycol. (II) At the same time, electron transfer occurs to exchange the glycol fragments in PET, an elimination reaction occurs, the chemical bond of the PET molecule is broken, and PET depolymerizes. (III) As the reaction proceeds, the degree of PET polymerization begins to decrease, and finally, the entire PET molecule is completely degraded to BHET monomer, while a certain amount of ethylene glycol is also generated.

## 4. Conclusion

A biomass-based catalyst was prepared from waste sunflower seed husk and applied to the catalytic alcoholysis of waste PET. The effects of reaction temperature, reaction time, catalyst dosage, and glycol dosage on the alcoholysis reaction were investigated. Based on one-factor experiment, a four-factor and three-level response surface experiment was designed according to the principle of BOX–Behnken experimental design, using the yield of BHET as the response value, and the optimal process conditions were determined: the alcoholysis temperature was 185 °C, the catalyst dosage was 0.89%, the reaction time was 4.9 h, and the glycol dosage was 14.6 ml, at which the actual yield of BHET was 79.57%, which was close to the value predicted by the simulation. In conclusion, it is of great significance that the sunflower seed husk matrix catalyst degrades waste PET in an environmentally friendly and efficient way, reduces pollution, promotes resource recycling, and contributes to sustainable development.

## Data availability

All data supporting the findings of this study are available within the paper and its ESI.†

## Author contributions

Guoliang Shen and Tiejun Xu conceived, planned, and supervised the experiments. Linlin Zhao and Haichen Wang were responsible for the main part of the experiment, including the preparation of the catalyst and its application in PET degradation. Ruiyang Wen and Sijin Jiang were responsible for the characterization and analysis of the catalyst. Linlin Zhao and Xiaocui Wang wrote the first manuscript, while all authors reviewed the manuscript.

## Conflicts of interest

The authors declare that they have no conflicts of interest.

## Supplementary Material

RA-015-D4RA07206E-s001

## References

[cit1] Zhou X., Lu X., Wang Q., Zhu M., Li Z. (2012). Pure Appl. Chem..

[cit2] Yue Q., Wang C., Zhang L., Ni Y., Jin Y. (2011). Polym. Degrad. Stab..

[cit3] Wang S., Wang C., Wang H., Chen X., Wang S. (2015). Polym. Degrad. Stab..

[cit4] Ryberg M. W., Hauschild M. Z., Wang F., Averous-Monnery S., Laurent A. (2019). Resour., Conserv. Recycl..

[cit5] Tsironi T. N., Chatzidakis S. M., Stoforos N. G. (2022). Packag. Technol. Sci..

[cit6] López-Fonseca R., Duque-Ingunza I., de Rivas B., Flores-Giraldo L., Gutiérrez-Ortiz J. I. (2011). Chem. Eng. J..

[cit7] Chen C. H. (2003). J. Appl. Polym. Sci..

[cit8] Imran M., Al-Masry W. A., Mahmood A., Hassan A., Haider S., Ramay S. M. (2013). Polym. Degrad. Stab..

[cit9] Nabid M. R., Bide Y., Fereidouni N., Etemadi B. (2017). Polym. Degrad. Stab..

[cit10] Fukushima K., Coulembier O., Lecuyer J. M., Almegren H. A., Alabdulrahman A. M., Alsewailem F. D., Mcneil M. A., Dubois P., Waymouth R. M., Horn H. W. (2011). J. Polym. Sci., Part A: Polym. Chem..

[cit11] Zhu M., Li S., Li Z., Lu X., Zhang S. (2012). Chem. Eng. J..

[cit12] Fang P., Liu B., Xu J., Zhou Q., Zhang S., Ma J. (2018). Polym. Degrad. Stab..

[cit13] Fukushima K., Jones G. O., Horn H. W., Rice J. E., Kato T., Hedrick J. L. (2020). Polym. Chem..

[cit14] Shukla S., Palekar V., Pingale N. (2008). J. Appl. Polym. Sci..

[cit15] Suo Q., Zi J., Bai Z., Qi S. (2017). Catal. Lett..

[cit16] Delle Chiaie K. R., McMahon F. R., Williams E. J., Price M. J., Dove A. P. (2020). Polym. Chem..

[cit17] Wang H., Yan R., Li Z., Zhang X., Zhang S. (2010). Catal. Commun..

[cit18] Veregue F. R., Pereira da Silva C. T., Moises M. P., Meneguin J. G., Guilherme M. R., Arroyo P. A., Favaro S. L., Radovanovic E., Girotto E. M., Rinaldi A. W. (2018). ACS Sustain. Chem. Eng..

[cit19] Chen F., Wang G., Li W., Yang F. (2013). Ind. Eng. Chem. Res..

[cit20] Liu B., Lu X., Ju Z., Sun P., Xin J., Yao X., Zhou Q., Zhang S. (2018). Ind. Eng. Chem. Res..

[cit21] Guo Z., Lindqvist K., de la Motte H. (2018). J. Appl. Polym. Sci..

[cit22] Imran M., Lee K. G., Imtiaz Q., Kim B.-k., Han M., Cho B. G., Kim D. H. (2011). J. Nanosci. Nanotechnol..

[cit23] Liu B., Fu W., Lu X., Zhou Q., Zhang S. (2018). ACS Sustain. Chem. Eng..

[cit24] Wang Q., Yao X., Geng Y., Zhou Q., Lu X., Zhang S. (2015). Green Chem..

[cit25] Wang Q., Geng Y., Lu X., Zhang S. (2015). ACS Sustain. Chem. Eng..

[cit26] Liu Y., Yao X., Yao H., Zhou Q., Xin J., Lu X., Zhang S. (2020). Green Chem..

[cit27] Liu M., Guo J., Gu Y., Gao J., Liu F. (2018). ACS Sustain. Chem. Eng..

[cit28] Al-Sabagh A. M., Yehia F. Z., Eissa A.-M. M., Moustafa M. E., Eshaq G., Rabie A.-R. M., ElMetwally A. E. (2014). Ind. Eng. Chem. Res..

[cit29] Yunita I., Putisompon S., Chumkaeo P., Poonsawat T., Somsook E. (2019). Chem. Pap..

[cit30] Kaiwen Z., Xuehang W., Wenwei W., Jun X., Siqi T., Sen L. (2013). Adv. Powder Technol..

[cit31] García J., López T., Álvarez M., Aguilar D., Quintana P. (2008). J. Non-Cryst. Solids.

[cit32] Ramachandran K., Sivakumar P., Suganya T., Renganathan S. (2011). Bioresour. Technol..

[cit33] Etim A. O., Musonge P., Eloka-Eboka A. C. (2020). Biofuels, Bioprod. Biorefin..

[cit34] Olatundun E. A., Borokini O. O., Betiku E. (2020). Renewable Energy.

[cit35] Alamu O., Waheed M., Jekayinfa S. (2007). Energy Sustainable Dev..

[cit36] Lukić I., Krstić J., Jovanović D., Skala D. (2009). Bioresour. Technol..

[cit37] Gohain M., Laskar K., Paul A. K., Daimary N., Maharana M., Goswami I. K., Hazarika A., Bora U., Deka D. (2020). Renewable Energy.

[cit38] Pathak G., Das D., Rajkumari K., Rokhum S. L. (2018). Green Chem..

