# Supplementary material for: Optimization of operating conditions for the catalytic alcoholysis of waste PET for the synthesis of BHET by sunflower seed husk matrix materials
Source: RSC Adv. 2025 Jan 2;15(1):207–15. doi: 10.1039/d4ra07206e (PMC11694505; doi:10.1039/d4ra07206e)
Supplement: RA-015-D4RA07206E-s001 [file RA-015-D4RA07206E-s001.pdf]

Supplementary Information (SI) for RSC Advances.
This journal is © The Royal Society of Chemistry 2024

Optimization of operating conditions for the catalytic alcoholysis of waste PET for the synthesis of BHET by sunflower seed husk matrix materials

Linlin Zhao, Guoliang Shen*, Ruiyang Wen, Tiejun Xu, Sijin Jiang, Xiaocui Wang, Haichen Wang

School of Petrochemical Engineering, Shenyang University of Technology, Liaoyang 111003, China

Corresponding author:

Guoliang Shen E-mail: shengl_shxy @sut.edu.cn

Table of contents

1. Effects of different roasting temperatures on the properties of prepared catalysts
2. Response surface design and results

1 Effects of different roasting temperatures on the properties of prepared catalysts

The effect of different roasting temperatures of sunflower seed shell catalyst on the yield of alcoholysis product BHET was investigated under the conditions of 1% catalyst (in terms of PET mass), reaction temperature 195 °C, reaction time 4 h, and ethylene glycol dosage 14 ml, and the results are shown in Fig. 1. It can be seen that the roasting of sunflower seed shells at a temperature of 750 °C had the optimum catalytic effect on PET, so the optimum roasting temperature of sunflower seed shells was 750 °C.

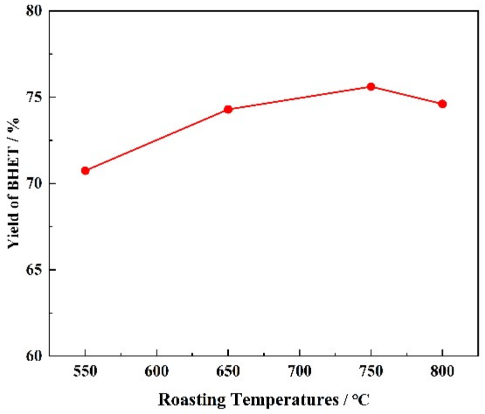

Fig.1 Effect of roasting temperature of sunflower seed husk on yield Results

2 Response surface design and results

Table S1 Response surface test design and results

serial number	considerations				BHET yield /%
	A	B	C	D	
1	-1	0	-1	0	75
2	0	0	0	0	79.3
3	1	0	-1	0	76
4	-1	0	1	0	76.7
5	0	-1	0	1	73.9
6	1	0	0	-1	77.7
7	0	1	1	0	79.6
8	0	0	1	-1	76.2
9	0	1	-1	0	74.3
10	-1	1	0	0	78.5
11	0	1	0	1	74.5
12	0	1	0	-1	79.4
13	0	-1	0	-1	78.1

14	0	0	0	0	78.1
15	-1	0	0	-1	76.5
16	0	0	-1	-1	73.2
17	1	0	1	0	76.5
18	0	1	-1	0	73.8
19	0	-1	1	0	78.8
20	1	-1	0	0	77.8
21	0	0	0	0	79.5
22	-1	0	0	1	76.2
23	0	0	1	1	73.1
24	1	1	0	0	75.3
25	0	0	0	0	79.8
26	0	0	0	0	78.3
27	-1	-1	0	0	75.2
28	1	0	0	1	76.8
29	0	0	-1	1	70.4
